# The microbial community, biogenic amines content of soybean paste, and the degradation of biogenic amines by *Lactobacillus plantarum* HM24

**DOI:** 10.1002/fsn3.2528

**Published:** 2021-10-26

**Authors:** Siyi Li, Xue Du, Lu Feng, Guangqing Mu, Yanfeng Tuo

**Affiliations:** ^1^ School of Food Science and Technology Dalian Polytechnic University Dalian China; ^2^ Dalian Probiotics Function Research Key Laboratory Dalian Polytechnic University Dalian China

**Keywords:** biogenic amine, correlation analysis, degrading ability, Microbial diversity, soybean paste fermentation

## Abstract

Soybean paste was a traditional fermented product in Northeast China, mainly fermented by molds, yeast, Bacillus, and lactic acid bacteria. This study investigated the dynamic changes of the microbial community and biogenic amine content during the fermentation of the traditional soybean paste. The microbial diversity of soybean paste in different regions was analyzed by MiSeq sequencing technology. The results showed that *Penicillium* and *Tetragenococcus* were the dominant microorganisms responsible for the fermentation of soybean paste. Biogenic amine was found in the traditional soybean paste at different fermentation stages, putrescine, and tyramine were the mainly biogenic amines and their content increased with the extension of fermentation time. *Serratia* in the soybean paste was positively correlated with the formation of spermine, cadaverine (*p* < .01), and β‐phenethylamine (*p* < .05), *Leuconostoc* was negatively correlated with tyramine formation (*p* < .05), and *Enterococcus* was positively correlated with the formation of histamine, tryptamine and cadaverine (*p* < .01). *Lactobacillus fermentum* HM22, *Lactobacillus plantarum* HM24, and *Enterococcus faecalis* YF10042 with strong biogenic amine degrading capacity were inoculated into the koji. After 20 days of fermentation, the degradation rates of tryptamine, β‐phenylethylamine, putrescine, cadaverine, histamine, and tyramine in soybean paste inoculated with *L. plantarum* HM24 were 35.31%, 43.14%, 30.18%, 33.44%, 32.74%, and 39.91%, respectively, indicating that the use of *L. plantarum* HM24 as a starter culture in soybean paste fermentation might be a good strategy for biogenic amines reduction.

## Graphical abstract

This study investigated the dynamic changes of the microbial community and biogenic amine content during the fermentation of the traditional soybean paste. The correlation between flora and bioamines and the degradation of biogenic amine by lactic acid bacteria were analyzed.

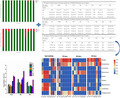

## 1 INTRODUCTION

The traditional fermented soybean paste in the northeast of China was made from soybeans and fermented naturally in the open air by natural microorganisms, and microbial community composition was an important factor in determining the flavor of soybean paste (Peng‐Fei et al., 2019; Yu & Sun, 2019). Yong et al. (2016) found that *Bacillus* and *Enterococcus* were the dominant microbes in the Korean soybean paste. Li et al. (2017) found that *Tetragenococcus*, *Lactobacillus*, *Staphylococcus*, *Acinetobacter*, *Pseudomonas,* and *Streptococcus* were the dominant bacteria responsible for the fermentation of doubanjiang. However, there were few reports on the dynamic changes of microorganisms and dominant fermenting bacteria in the whole fermentation stage of soybean paste. Therefore, it was of great significance to deeply understand the changes of microbial communities at different fermentation stages of traditional soybean paste. In recent years, MiSeq sequencing technology has been widely used, given its accuracy and sequencing depths, to determine the accurate microbial community and dynamic change in the microbial community during fermentation of soybean paste (Jeong et al., 2016; Nam et al., 2012).

Fermented soybean products contained abundant proteins and biogenic amine precursor amino acids. If microorganisms with decarboxylase activity were present simultaneously in the fermentation process, biogenic amine would be produced in the natural fermentation process of soybean paste. It was reported that tyramine content was generally higher in protein‐rich fermented products (Awan et al., 2008; Fausto et al., 2016; Mayer & Gregor, 2018; Mah et al., 2019). Typical biogenic amines included tryptamine, β‐phenethylamine, putrescine, cadaverine, histamine, tyramine, spermidine, and spermine (Awan et al., 2008; Lu et al., 2018; Shukla et al., 2010), among which histamine and tyramine had relatively high toxicity (Zhang et al., 2013; Taylor et al., 1978). Intake of high concentration biogenic amine may cause headache, palpitation, spasm, vomiting, and pupil dilation in some people (Hazards, 2011; Rice et al., 1976; Lehane & Olley, 2000). Biogenic amines have been found in fermented soybean products such as sufu (Guan et al., 2013), soy sauce (Lu et al., 2019), Japanese miso (Shukla et al., 2011), and natto (Kim et al., 2012). However, there were few systematic studies on the content and variation of biogenic amines in traditional soybean paste in northeast China.

Due to the unique flavor, traditional fermented soybean paste was very popular in northeast China. However, few studies were conducted on the correlation between dynamic changes of microorganisms and biogenic amine content during the soybean paste fermentation. In this study, the microbial community changes during the soybean paste fermentation, made by families in Heilongjiang Province and Liaoning Province, were studied by high‐throughput sequencing method and the content of biogenic amines at different fermentation stages was detected by high‐performance liquid chromatography, to clarify the correlation between dynamic changes of microorganisms and the content of biogenic amines. The strains with the ability to degrade biogenic amine were screened and applied in lab‐scale soybean paste fermentation.

## 2 MATERIALS AND METHODS

### 2.1 Preparation of fermented soybean paste and sample collection

The soybean paste in this study was homemade by some families in northeast China. The soybean paste was prepared just as follows. Raw soybeans were soaked in water at room temperature for 24 h and then cooked in boiling water for one hour. After cooling, drained and mashed soybeans into soybean cake and placed in a position with sufficient sunlight and good ventilation at room temperature for 10–15 days. When fungi mycelium appeared in the soybean cake, the cake was smashed and immerged into 12% brine, which was agitated daily.

Twelve soybean paste samples prepared by families in the northeast of China were collected. The soybean paste samples were collected in four different fermentation phase (a, b, and c of the soybean paste with fermentation time of 0, 20, 40, and 60 days). Approximately 5 g of each sample was collected in an aseptic pipe, and each sample was collected in three replicates and stored at −20°C for further analysis. The samples were assigned as koji a, 20a, 40a, and 60a, koji b, 20b, 40b, and 60b, and koji c, 20c, 40c, and 60c. The twelve samples were used for the determination of microbial diversity and the content of biogenic amines.

### 2.2 Gene sequencing

The genomic DNA was extracted from the soybean paste samples with the Illumina Truseq DNA kit following the manufacturer's instructions. The purity of the genomic DNA was confirmed by 1% agarose gel electrophoresis. Primers 338F (ACTCCTACGGGAGGCAGCAG) and 806R (GGACTACHVGGGTWTCTAAT) were designed according to the V3–V4 region of bacterial 16S rRNA gene. Primers ITS1F (CTTGGTCATTTAGAGGAAGTAA) and ITS2R (GCTGCGTTCTTCATCGATGC) were designed based on the ITS1F‐ITS2R region of the fungal internal transcribed spacer (Li, Tang, et al., 2019; Li, Zou, et al., 2019) and used the following reaction conditions: an initial denaturation at 95℃ for 3 min, followed by 35 cycles at 95℃ for 30 s, 55℃ for 30 s, and 72℃ for 45 s and a final 10 min extension at 72℃. The PCR product was sequenced by Shanghai Majorbio Bio‐pharm Technology Co., Ltd.

### 2.3 Sequencing and data analysis

After PCR and purification, a DNA library was constructed and run on the Miseq Illumina platform (Illumina, San Diego) according to the standard protocols by Majorbio Bio‐Pharm Technology Co. Ltd.

### 2.4 Biogenic amines (BAs) contents determination by HPLC

Eight kinds of biogenic amine standard solutions with a concentration of 1000 mg/L were prepared in 0.1 M hydrochloric acid solution. Take 750 μl of biogenic amine standard solution, add an equal volume of dansyl chloride and 150 μl of saturated sodium carbonate solution, incubated at 45℃ for 30 min, and then add 150 μl of ammonia water and incubated at 45℃ for 15 min. The derivatized biogenic amine standard solutions were stored at 4℃ until the use. All standard chemicals were obtained from Shanghai yuanye Bio‐Technology Co., Ltd.

Accurately weigh 5.00 g samples, add 20ml 5% trichloroacetic acid solution, vibratory extraction for 30 min, centrifuged at 8000 g at 4℃ for 5 min, and collected filtrate, the residues were mixed with trichloroacetic acid and centrifuged again. The supernatant was combined with the previously collected supernatant and adjusted to a volume of 50 ml with 5% trichloroacetic acid and took 10.00 ml of the above extract and added an equal volume of n‐hexane, vortexed for 5 min discarded the upper organic phase. The 750 μl of the extract was transferred to a tube capped with a stopper, added 150 μl of saturated sodium carbonate solution, and 750 μl of dansyl chloride. The mixed solution was incubated at 45℃ for 30 min for derivatization. Finally, added 150 µl ammonia water and incubated at 45℃ for 15 min (Lu et al., 2018; Li, Tang, et al., 2019; Li, Zou, et al., 2019; Yu & Sun, 2019).

The sample and biogenic amine standard solutions were filtered through a 0.22μm syringe filter and analyzed by HPLC equipped with a Zorbax Eclipse XDB‐C18 column (4.6 mm × 250 mm, 5 µm) at 254 nm. The flow rate was 1.0 ml/min, and the column oven temperature was 40℃ (Lu et al., 2018; Yu & Sun, 2019). Acetonitrile and water were used as mobile phases, and a gradient mode was programmed shown in Table 1 (Li, Tang, et al., 2019; Li, Zou, et al., 2019; Yu & Sun, 2019).

**TABLE 1:** The gradient elution program

Elution time/min	Mobile phase A/%	Mobile phase B/%
0	45	55
10	45	55
15	35	65
20	20	80
25	20	80
30	10	90
33	10	90
35	45	55

### 2.5 Evaluation of BA‐degradation Ability

To evaluate the BAs‐degradation ability, three strains of bacteria selected from laboratory‐collected strains were inoculated into MRS broth with 100 mg/L of tryptamine, β‐phenylethylamine, putrescine, cadaverine, histamine, tyramine, spermidine, and spermine and incubated at 37℃ for 48 h (Kung et al., 2017). Cultures of each strain were harvested by centrifugation at 4000 g for 10 min, the MRS without *Lactobacillus* inoculation was used as the control, determined the concentration of BAs in the culture medium by HPLC after 48 hr of incubation at 37℃. The value of BA‐degradation rate was determined based on an equation:

X=[(c_1_‐c_2_)/c_1_] ×100%, among them, *X*: degradation ability of strains, %; *C_1_*: BAs concentration of control group, mg/L; *C_2_*: BAs concentration of experimental group, mg/L.

### 2.6 Identification of BA‐degradation bacterial strains

The strains with BA‐degradation ability were identified through 16 S ribosomal DNA sequencing analysis. The 16 S ribosomal DNA was obtained through PCR amplification using the following primers: 338F (ACTCCTACGGGAGGCAGCAG) and 806R (GGACTAC HVGGGTWTCTA A T). The PCR product was sequenced by Shanghai Majorbio Bio‐pharm Technology Co., Ltd.

### 2.7 Soybean paste fermentation with BAs degradation strains

Soybeans were crushed after high‐pressure steaming for 20 min, and koji was made naturally in the laboratory environment for two months. The koji was ground and put into water containing 8% sea salt, and mixed koji and brine in the ratio of 1:1 (Xu et al., 2020). To test the effect of isolated bacteria strains on the degradation of BAs in soybean paste, added 4% bacterial solution into the soybean paste. The soybean paste without inoculation of the above bacteria solution was adopted as control. Sealed the bottle with six layers of gauze and fermented in natural environment. During soybean paste fermentation, samples were collected on different fermentation days. BAs concentrations, pH, total acid, amino acid nitrogen, and reducing sugar of the collected samples were analyzed and compared.

### 2.8 Statistical analysis

All analysis experiments were conducted at least three replicates. The difference significance was analyzed by one‐way ANOVA method using the statistical software SPSS 20.0, and mean values were compared by Tukey's HSD test at 5%.

## 3 RESULTS AND DISCUSSION

### 3.1 Dynamics of microbial community during soybean paste fermentation

#### 3.1.1 Analysis of diversity indices of bacterial and fungi sequences

**FIGURE 1:**
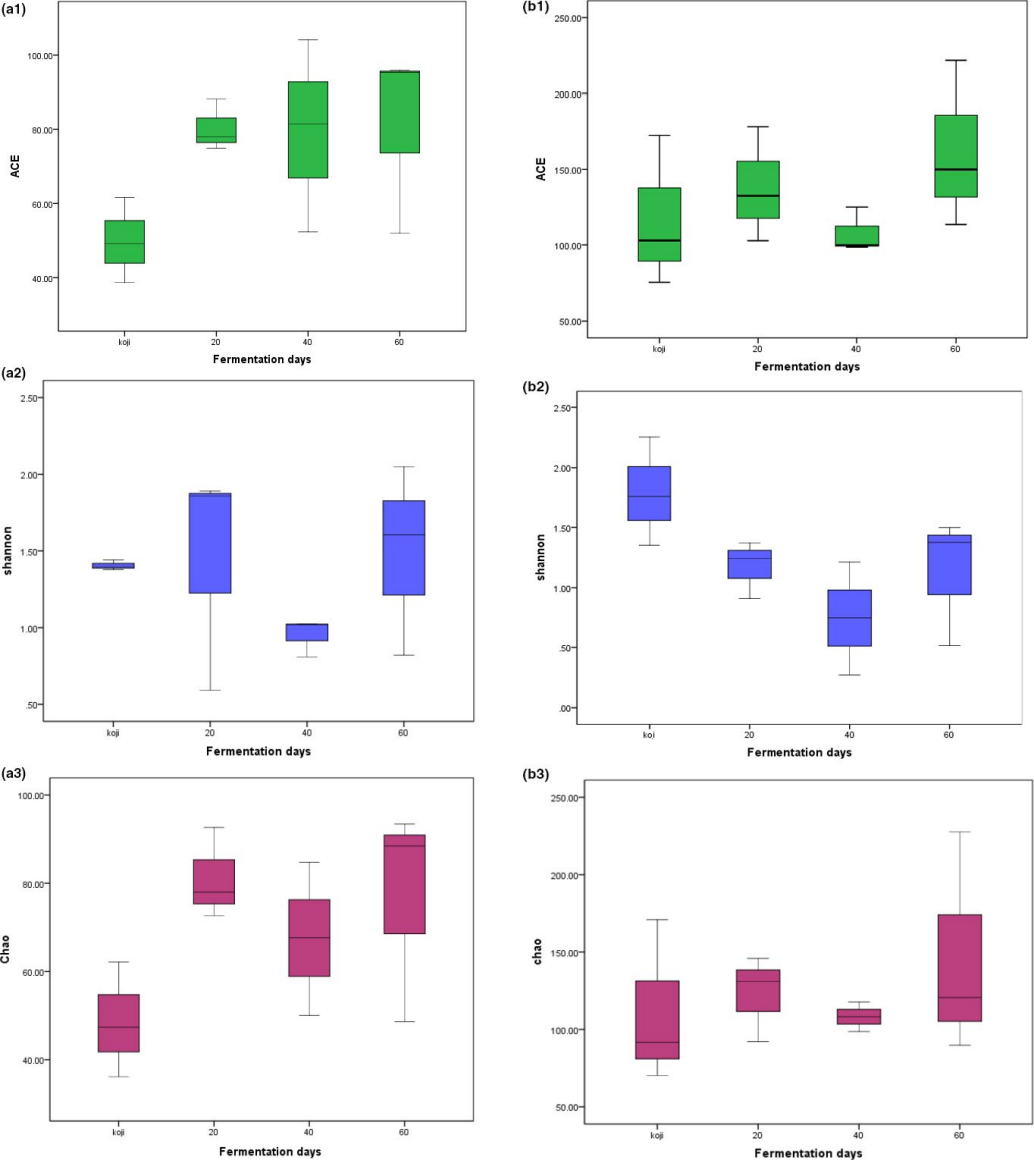
Diversity index box figure of bacteria and fungi in northeast farmhouse soybean paste at different fermentation times. (a1) ACE index in bacteria, (a2) Shannon index in bacteria, (a3) Chao index in bacteria, (b1) ACE index in fungi, (a2) Shannon index in fungi, (a3) Chao index in fungi

Shannon index, Chao, and abundance‐based coverage estimator (ACE) showed species diversity and richness (Fang et al., 2015). The diversity indices values of the soybean paste samples from northeast China in Figure 1 showed that the samples fermented for 60 days had the richest genera abundance and diversity in terms of bacteria and fungi, followed by the samples fermented for 20 days. The bacterial diversity of samples at the koji‐making stage was higher than that of the samples fermented for 20 days. Samples fermented for 40 days had the lowest species richness and diversity.

#### 3.1.2 Analysis of bacterial and fungal community composition in soybean paste

The fermentation of soybean paste involved a great diversity of microorganisms, including bacteria and fungi (Sun et al., 2018). The preparation of soybean paste from northeast China was attributed to the interaction of a variety of microorganisms under natural conditions. This natural fermentation method could form a unique microbial community in the soybean paste. Many fungi, such as *Cladosporium*, *Aspergillus*, *Eurotium*, *Mucor*, *Lichtheimia*, *Penicillium*, *Scopulariopsis,* and *Rhizopus*, were found in naturally fermented soybean pastes (Hong et al., 2011, 2012; Kim et al., 2013). *Aspergillus*, *Oryzae,* and *Yeast* species were found in fermented soybean paste in Shandong province, showing the diversity of fungi in soybean paste (Gao et al., 2013).

**FIGURE 2:**
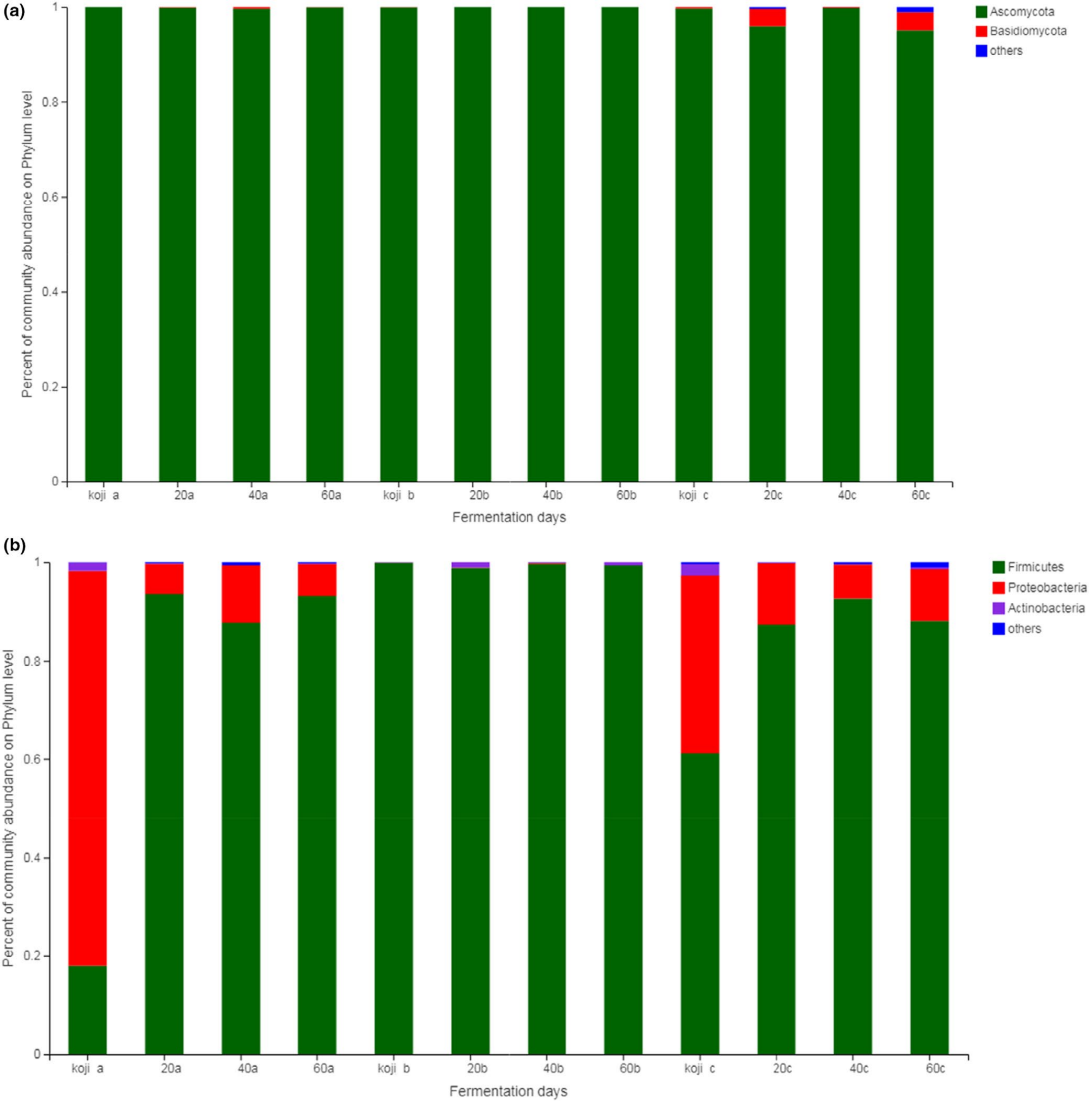
Microbial community in soybean paste samples at the phylum level. (a) Microbial community of three samples of a, b, and c in fungi, (b) Microbial community of three samples of a, b, and c in bacteria

**FIGURE 3:**
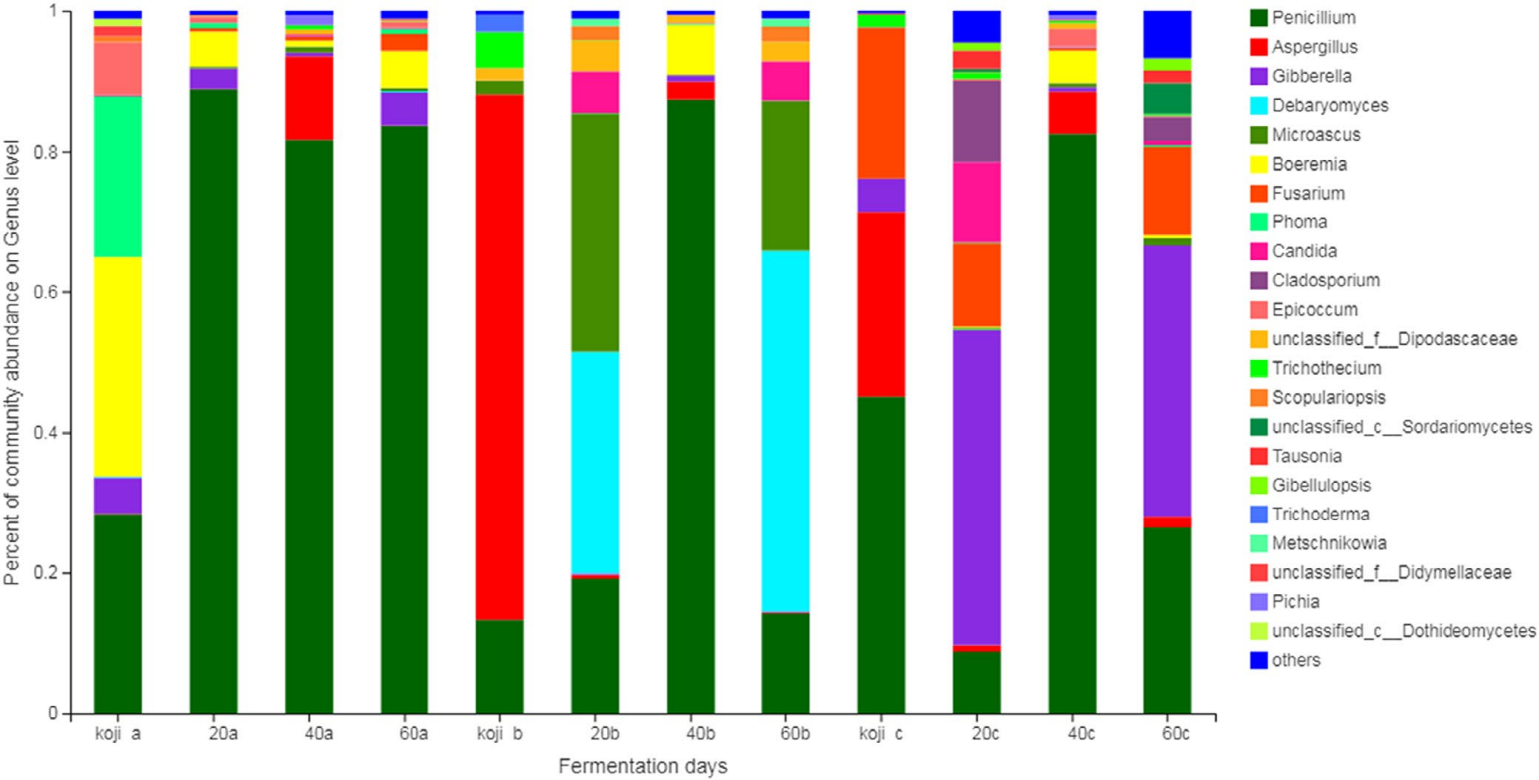
Composition of fungal community in soybean paste samples at genus level

**FIGURE 4:**
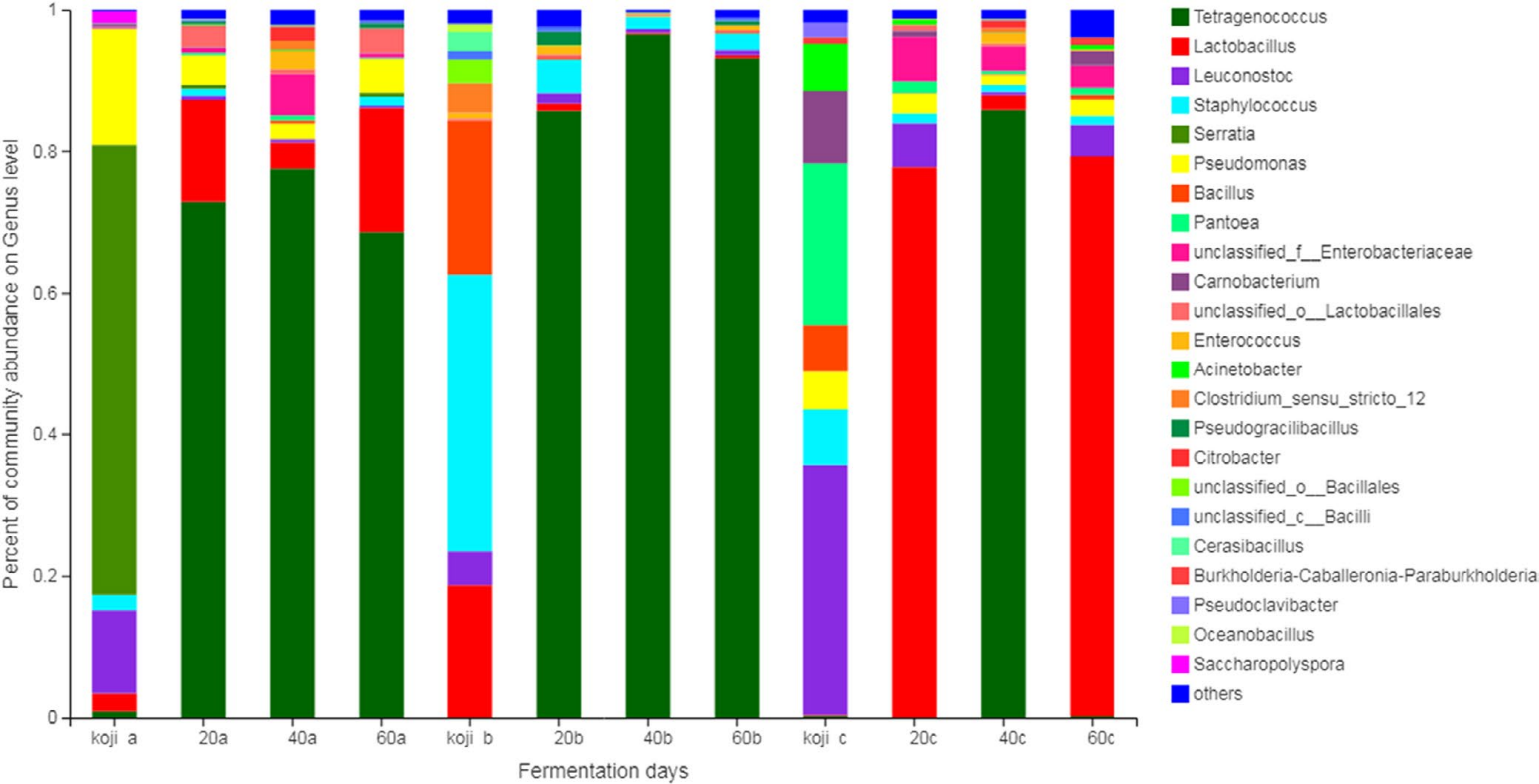
Bacterial community composition in soybean paste samples at genus level

The relative abundance of bacterial and fungal in soybean paste at the phylum level was shown in Figure 2. *Ascomycota* was dominant in all samples regardless of fermentation time, with the proportion ranging from 94.96% to 99.99%. *Firmicutes* was the dominant fermenting bacteria. Both sample a and sample c contained *Proteobacteria* and *Actinobacteria*, especially in the koji‐making stage of sample a, the proportion of *Proteobacteria* reached 80.29%. Li et al. (2017) found that *Firmicutes* and *Proteobacteria* were predominant in Chinese traditional fermented broad bean. As shown in Figures 3 and 4, *Aspergillus*, *Penicillium,* and *Gibberella* were present in the soybean paste samples at the koji‐making stage. *Aspergillus* and *Penicillium* were the main strains in the early fermentation stage. During the 60‐day fermentation of soybean paste, the proportion of *Aspergillus* decreased gradually, *Penicillium* increased and became the dominant fermentation strain.

The proportion of *Tetragenococcus* in samples increased significantly. When the fermentation time was 40 days, the proportion of *Tetragenococcus* in samples was 96.54%, which became the dominant bacteria in the later fermentation period of soybean paste. *Tetragenococcus*, belonging to halophilic lactic acid bacteria, was often present in fermented food as the major population such as Doenjang (Kim et al., 2009) and soy sauce (Zhang et al., 2013). With the extension of fermentation time, the proportion of *Bacillus* decreased, the proportion of *Lactobacillus* decreased firstly and then increased, and the proportion of *Lactobacillus* reached 77.71% after fermented for 20 days. Petersen et al. (2011) reported that the lower pH in the fermentation system was harmful to the growth of *Bacillus*, leading to the gradual decline of *Bacillus*. With the increase of fermentation, *Tetragenococcus* and *Penicillium* became dominant bacteria in the soybean paste.

### 3.2 Contents of biogenic amines in soybean paste samples in different fermentation phase

**FIGURE 5:**
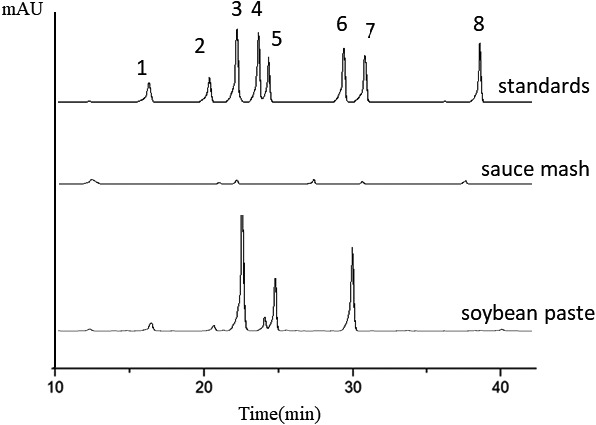
Chromatogram of derivatives of biogenic amines standards and sample (1) Tryptamine, (2) β‐phenylethylamine, (3) putrescine, (4) cadaverine, (5) histamine, (6) tyramine, (7) spermidine, and (8) spermine. The peaks that were omitted have no meaning. As shown, the peaks of the 8 standard derivatives were shown later and flagged as 1, 2, 3, 4, 5, 6, 7, and 8

Through the elution procedure, each BAs was detected in turn. The representative high performance liquid chromatography chromatogram and peak identification recorded for mixer of standard BAs and a fermented soybean paste samples were shown in Figure 5. The biogenic amines contents in the soybean paste samples from northeast China were determined by high‐performance liquid chromatography. The biogenic amines content in 12 soybean paste samples was shown in Table 2. In the koji‐making stage, the contents of the eight biogenic amines and the total biogenic amines were significantly lower than those in other fermentation phases. The content of total biogenic amines in sample a and sample b after fermented for 60 days was significantly higher than that in sample c (*p* < .05).

**TABLE 2:** Contents of Biogenic Amines in traditional northeast soybean paste Samples

BAs mg/kg	Try	β‐Phe	Put	Cad	His	Tyr	Spd	Spm	BAs
Time/day									
(a) The changes of biogenic amines in northeast soybean paste with different fermentation time in sample a									
koji	ND	23.5 ± 1.77	61.5 ± 1.71	ND	ND	16.01 ± 1.33	ND	7.3 ± 0.11	108.31 ± 4.92
20	299.10 ± 2.80	141.61 ± 9.92	973.14 ± 16.82	129.4 ± 3.34	744.46 ± 13.1	995.61 ± 3.58	ND	28.1 ± 1. 98	3311.42 ± 51.54
40	284.16 ± 4.11	154.72 ± 3.71	1021.9 ± 17.12	132.24 ± 9.75	773.47 ± 10.3	1023.72 ± 9.43	ND	59.1 ± 8.41	3449.31 ± 62.83
60	315.92 ± 2.18	233.47 ± 1.89	1027.5 ± 7.56	134.19 ± 1.92	813.72 ± 7.56	1075.37 ± 11.91	ND	70.34 ± 1.94	3670.51 ± 34.96
(b) The changes of biogenic amines in northeast soybean paste with different fermentation time in sample b									
koji	21.71 ± 2.04	17.82 ± 0. 87	28.11 ± 0.91	10.54 ± 0.61	17.7 ± 1.81	21.20 ± 1.12	18.41 ± 0.59	ND	135.49 ± 7.95
20	137.81 ± 2.81	22.54 ± 0.78	621.62 ± 5.62	24.40 ± 0.51	349.76 ± 3.76	255.91 ± 3.83	4.22 ± 0.31	ND	1416.26 ± 17.62
40	209.92 ± 1.33	37.54 ± 0.75	726.63 ± 3.68	30.98 ± 0.91	446.22 ± 1.89	355.32 ± 2.37	5.70 ± 0.96	ND	1812.31 ± 11.89
60	197.84 ± 5.63	34.76 ± 0.49	705.92 ± 1.78	29.12 ± 1.06	450.86 ± 7.28	356.23 ± 0.63	5.85 ± 0.36	ND	1780.58 ± 17.67
(c) The changes of biogenic amines in northeast soybean paste with different fermentation time in sample c.									
koji	ND	ND	74.57 ± 0.47	ND	ND	ND	55.01 ± 1.26	ND	129.58 ± 1.73
20	257.62 ± 7.44	29.43 ± 1.61	23.20 ± 0.54	24.53 ± 0.49	32.42 ± 1.32	20.46 ± 0.91	5.44 ± 1.23	ND	393.10 ± 13.54
40	263.51 ± 9.36	31.84 ± 0.93	23.44 ± 0.63	25.37 ± 0.23	36.32 ± 2.54	23.02 ± 3.11	6.90 ± 0.42	ND	410.40 ± 17.22
60	273.93 ± 2.28	33.61 ± 1.34	24.82 ± 0.93	26.43 ± 0.92	40.82 ± 2.73	25.41 ± 1.12	5.24 ± 0.12	ND	430.26 ± 9.44

Note

Values are expressed as averages of three independent experiments ±*SD*. ND indicates that no biogenic amines have been detected.

In sample a and sample b, the contents of Put, His, and Tyr were the highest. The highest content of Try was found in sample c, and the other biogenic amines were lower. European Food Safety Authority (Hazards, 2011) recommended that the intake of His and Tyr should not surpass 50 and 600mg per meal, respectively. Brink et al. (1990) recommended that the levels of His, Tyr, and Phe in food should be lower than 100, 800, and 30 mg/kg, respectively.

Excessive intake of biogenic amines may be harmful to human being health, including blood pressure changes, vomiting, palpitations, breathing disorders, headaches, and other symptoms (Hazards, 2011; Rice et al., 1976; Shukla et al., 2010). In this study, biogenic amine was commonly found in the traditional soybean paste. Sample a and sample b were produced by families in Heilongjiang Province, and sample c was produced by families in Liaoning Province. Different fermentation technology and environments may result in some differences in the biogenic amine content among these soybean paste samples. The formation of biogenic amines depended primarily on fermentation conditions and microbes (Linares et al., 2011; Kalac & Krausova, 2005; Spano et al., 2010). When the microbes with amino acid decarboxylase propagate in food rich in protein and free amino acids, biogenic amines could accumulate in the foods. Some kinds of biogenic amines were detected in the traditional fermented soybean paste from northeast China, which was naturally fermented by *Aspergillus oryzae*, *yeast*, *Lactobacillus,* and other microorganism (Li et al., 2014; Yu & Sun, 2019). Traditional fermented soybean paste had a complex and diverse microbial community, rich free amino acids and was easy to accumulate biogenic amines (Alvarez & Moreno‐Arribas, 2014). Therefore, it was important to detect the type and content of biogenic amines and develop effective methods to reduce biogenic amines in soybean paste (Gong et al., 2014; Santos, 1996; Yu & Sun, 2019).

### 3.3 Correlation analysis of microbiota and biogenic amine content in samples

**FIGURE 6:**
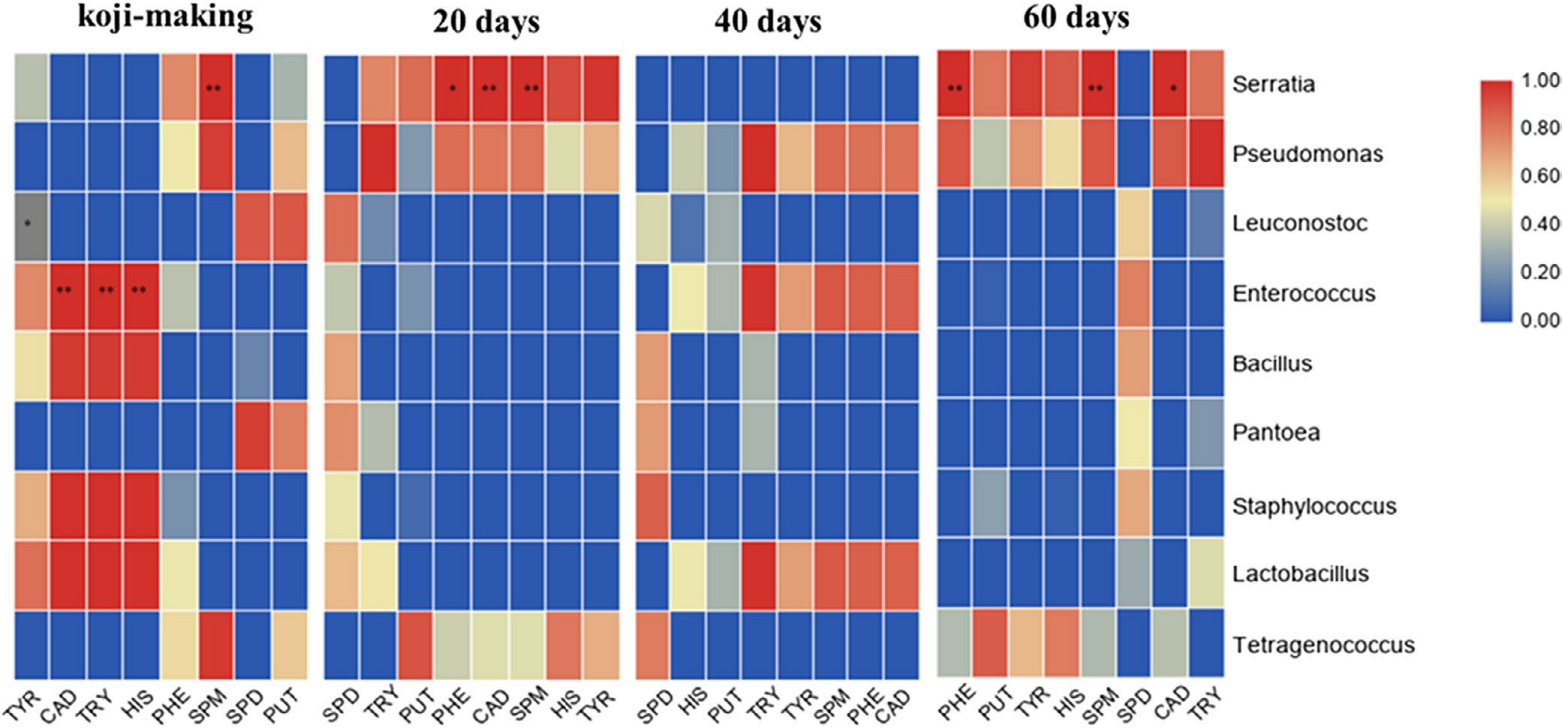
Correlation analysis of bacteria and biogenic amine content in northeast soybean paste. **p < .01; *p < .05

The amount of biogenic amines formed in fermented food products was greatly influenced by the food composition, microflora, and other factors (Carelli et al., 2008). Peng‐Fei et al. (2019) found that bacteria had a stronger effect on biogenic amines than fungi in da‐jiang. We investigated the correlation between bacterial community composition and biogenic amine content in different fermentation periods of the soybean paste from northeast China. As shown in Figure 6, *Serratia* was positively correlated with the formation of Spm in the koji‐making stage (*p* < .01), and also positively correlated with the formation of Spm, Cad (*p* < .01), and β‐Phe (*p* < .05) in the samples fermented for 20 days. There was a negative correlation between *Leuconostoc* and the formation of Tyr (*p* < .05), and *Enterococcus* was positively correlated with His, Try, and Cad (*p* < .01).

In this study, the total biogenic amine content of sample a was the highest, followed by sample b, and sample c had the lowest total amine content. The results of flora analysis showed that sample a contained high levels of *Serratia* and *Enterococcus*. *Serratia* belongs to the *Enterobacteriaceae*, which showed high lysine decarboxylase and ornithine decarboxylase activities, and could produce a high concentration of cadaverine and putrescine (Linares et al., 2011). Jeon et al. (2018) believed that the high concentration of tyramine in Cheonggukjang was related to *Enterococcus*, and other studies have reported that *Enterococcus* in fermented foods had tyrosine decarboxylase (Kang et al., 2017). Sample c contained 35.22% of *Leuconostoc* in the koji‐making stage, and all of the later fermentation stages contained *Leuconostoc*. Spearman correlation analysis showed that *Leuconostoc* was negatively correlated with the contents of tyramine (Rollan et al., 1995), which may explain the lowest content of biogenic amine in sample c.

### 3.4 Isolation and identification of strains with biogenic amines degrading ability

**FIGURE 7:**
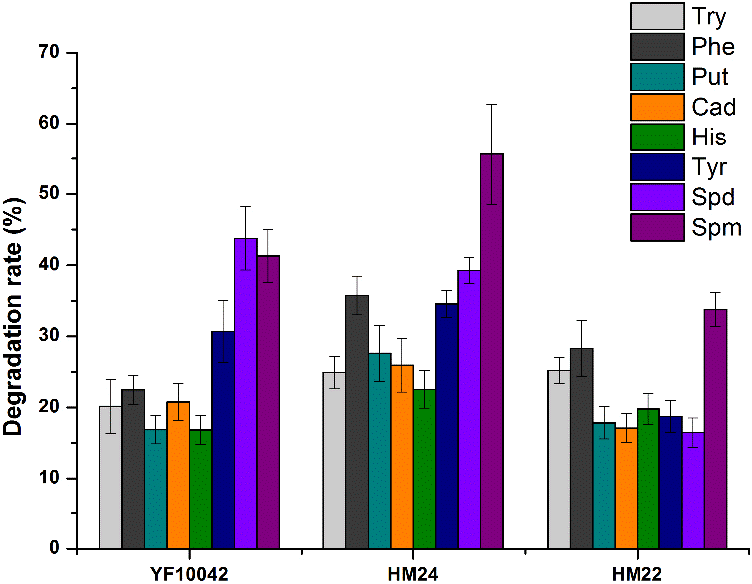
Degradation of Lactic Acid Bacteria by Strains

The biogenic amines degrading ability of the strains HM22, HM24, and YF10042 were shown in Figure 7. The degrading ability of HM24 to spermine and histamine was significantly higher than that of the other strains. HM22, HM24, and YF10042 were identified based on 16 S ribosomal DNA sequencing analysis. HM22 strain had a 99% identity with *Lactobacillus fermentum*, HM24 strain had a 100% identity with *Lactobacillus plantarum* while YF10042 strain had a 100% identity with *Enterococcus faecalis*. HM22 strain was identified as *Lactobacillus fermentum*, HM24 strain was identified as *Lactobacillus plantarum* while YF10042 strain was identified as *Enterococcus faecalis*.

### 3.5 Application of the strains with biogenic amine degrading ability in soybean paste fermentation

**FIGURE 8:**
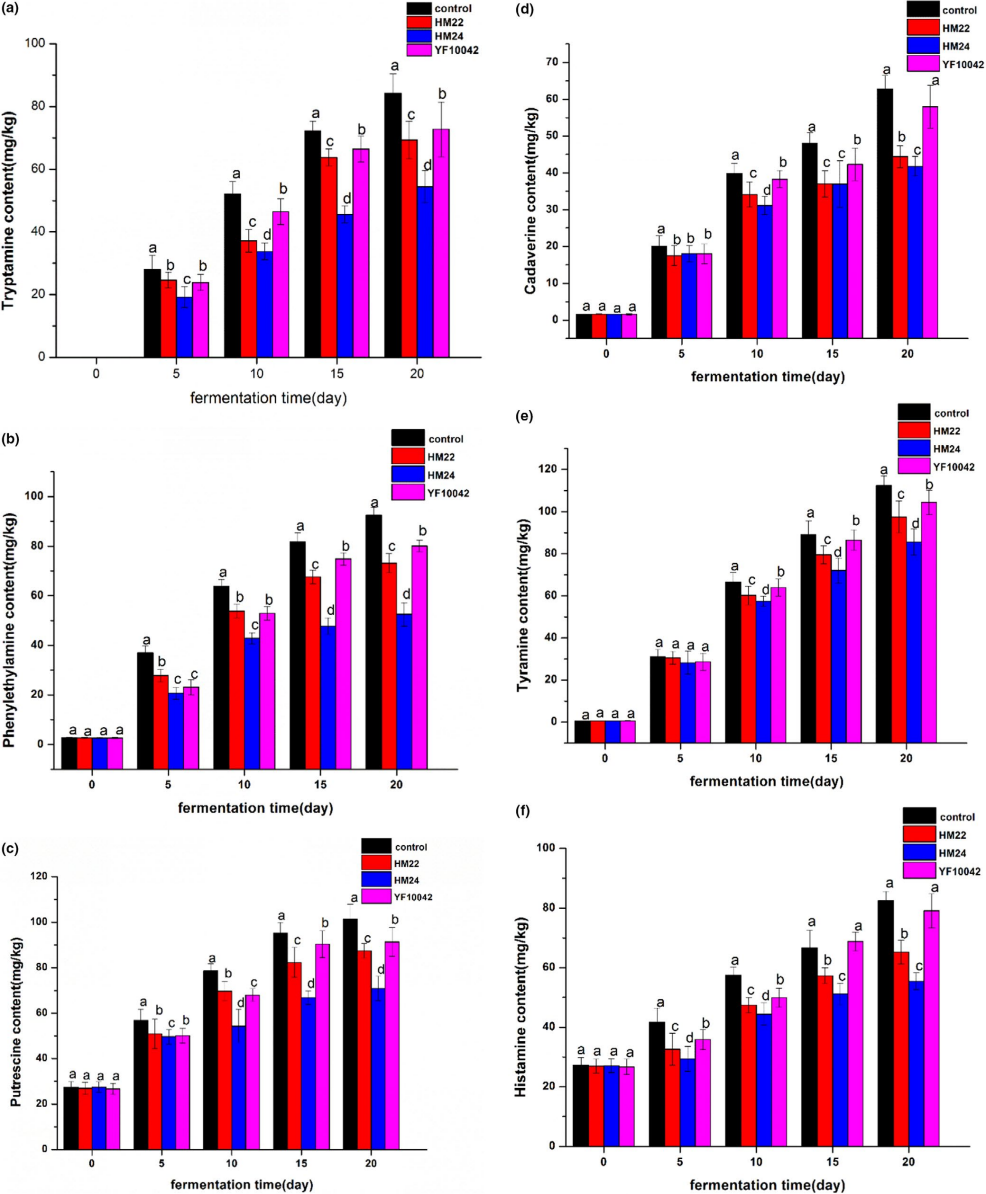
Effect of biogenic amine degrading strain on biogenic amine in soybean paste. (a) tryptamine, (b) β‐phenethylamine, (c) putrescent, (d) cadaverine, (e) histamine, (f) tyramine

The three strains HM22, HM24, and YF10042 with biogenic amines degrading ability were applied in the fermentation of lab‐scale soybean paste. As shown in Figure 8a, the content of tryptamine in soybean paste inoculated with the three strains showed reduction after fermentation for 5–20 days, among which the reduction by *Lactobacillus plantarum* HM24 was the most significant. After fermentation for 20 days, the content of tryptamine in the *Lactobacillus plantarum* HM24 group was reduced by 35.31% compared to the control. It can be seen from Figure 8b–f that after 20 days of fermentation by *Lactobacillus plantarum* HM24, the contents of tyramine, β‐phenethylamine, putrescine, cadaverine, and histamine in the treated group were significantly lower than those in the control. These results indicated that *Lactobacillus plantarum* HM24 showed better degradation of tryptamine, tyramine, putrescine, cadaverine, β‐phenethylamine, and histamine in the fermented soybean paste.

**FIGURE 9:**
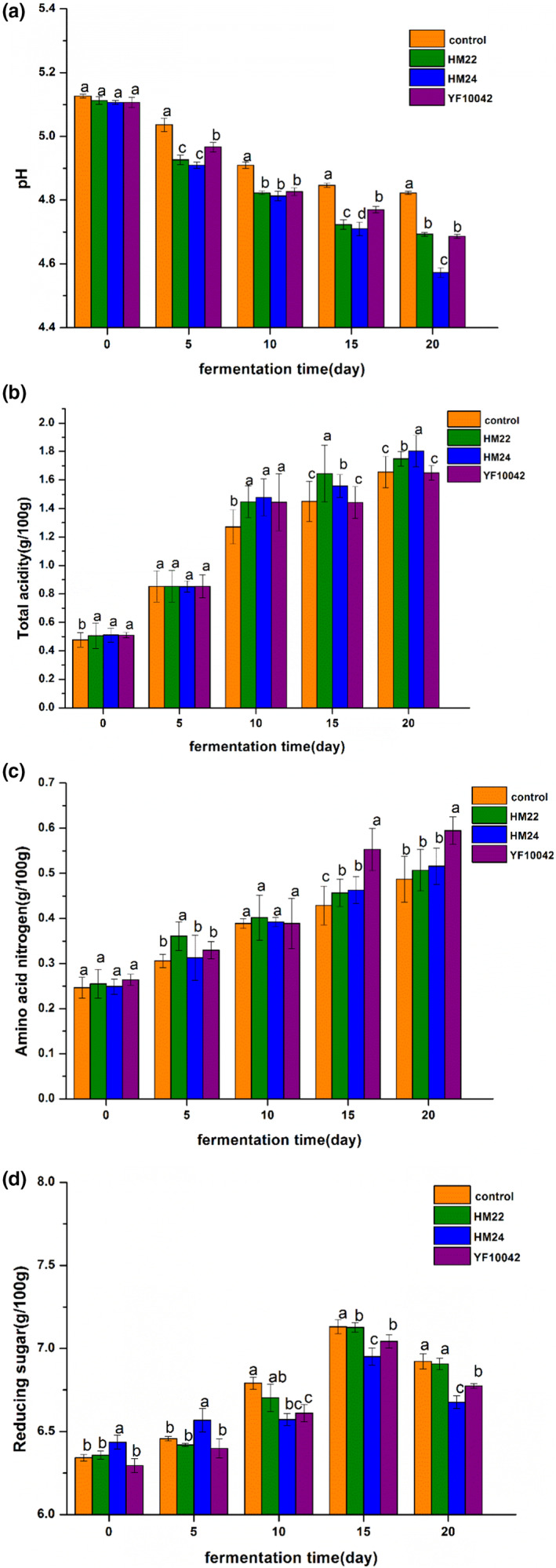
Effect of biogenic amine degrading strain on pH, total acid, amino acid nitrogen, and reducing sugar content. (a) pH, (b) total acid content, (c) amino acid nitrogen content, (d) reducing sugar content

As shown in Figure 9a, the pH value of each group showed a trend of decline during the fermentation period, and the pH of the HM24 group was significantly lower than that of the other groups. The low pH value of fermented soybean paste was related to the growth of lactic acid bacteria and the production of organic acids from free sugars (Chun et al., 2019). The content and variation of total acid and amino acid nitrogen had an important effect on the quality of soybean paste, so the fermentation process of Douchi can be optimized by the change of amino nitrogen content and acidity (Li, Tang, et al., 2019; Li, Zou, et al., 2019). Figure 9b and c showed that the total acid content maintained an increasing trend and then stabilized, the content of amino acid nitrogen increased continuously. The total acid and amino acid nitrogen content of the control was significantly lower than the lactic acid bacteria treated group, which was similar to the trend of total acid and amino acid nitrogen in douchi (Yu & Sun, 2019). Figure 9d showed that the content of reducing sugar firstly increased and then decreased, which was the same as the results of Tang et al. (2013). The reason may be that the amylase, cellulase, and saccharifying enzyme produced by mold in soybean paste hydrolyze carbohydrates to produce reducing sugar. In the later stages of fermentation, the pH and total acid inhibited the activity of amylase, and reducing sugars are consumed by microbial growth and Maillard reaction, which led to the decrease of reducing sugar (Tang et al., 2013). The reducing sugar content of the HM24 treated group was significantly lower than those of the other groups during fermentation for 10 to 20 days, indicating that HM24 could grow well in soybean paste.

## 4 CONCLUSION

In this study, the microbial diversity of traditional fermented soybean paste in northeast China was investigated in terms of both bacteria and fungi. The fermentation of homemade soybean paste was attributed to the combination of bacteria and fungi. *Tetragenococcus* and *Penicillium* were the dominant bacteria in the fermentation process of soybean paste. With the extension of fermentation time, the content of biogenic amine in the soybean paste gradually increased, the highest value of biogenic amine was 3670.51 ± 34.96 mg/kg, among which tyramine (Tyr) and putrescine (Put) were higher. It was found that spermine (Spm), cadaverine (Cad), and β‐phenylethylamine (β‐Phe) were positively correlated with *Serratia*, whereas tyramine (Tyr) was negatively correlated with *Leuconostoc*. *Lactobacillus plantarum* HM24 exhibited strong degradation of histamine (His), β‐phenethylamine (β‐Phe), and tyramine (Tyr) during the fermentation of soybean paste, indicating that the use of *Lactobacillus plantarum* HM24 as starter culture reduced the production of biogenic amine.

## AUTHOR CONTRIBUTIONS

**Siyi Li:** Conceptualization (equal); Data curation (equal); Formal analysis (equal); Funding acquisition (equal); Investigation (equal); Methodology (equal); Project administration (equal); Resources (equal); Software (equal); Supervision (equal); Validation (equal); Visualization (equal); Writing‐original draft (equal); Writing‐review & editing (equal). **Xue Du:** Data curation (equal); Methodology (equal). **Guangqing Mu:** Resources (equal).

## Data Availability

The datasets used or analyzed during the current study are available from the corresponding author on reasonable request.
